# Reasons for non-participation in a randomised controlled trial and the effect of audiovisual material

**DOI:** 10.1186/1745-6215-16-S2-P112

**Published:** 2015-11-16

**Authors:** Neil Nathwani, Amanda Davis, Evgenia Konstantakopoulou, Gus Gazzard

**Affiliations:** 1 Moorfields Eye Hospital, London, UK

## Background

Recruitment in randomised controlled trials (RCT) has been the topic of research for many years. This study, nested within the NIHR Laser in Glaucoma and Ocular Hypertension (LiGHT) Trial, looks into the main reasons for non-participation in a Trial investigating the quality of life and cost-effectiveness of two established glaucoma treatments, and whether the use of audiovisual material (video) improves recruitment rates.

## Methods

Prospective participants to the LiGHT Trial were approached by a member of the research team and were given an explanation of the Trial. Patients who refused to participate were asked to explain the reason for their refusal choosing from a pre-populated list of 12 items and were also given the option to add other reasons. Fifteen months into the Trial a video was also used to facilitate recruitment. The material of the video was the same as delivered by the recruiting team, but was delivered by the Principal Investigator (PI).

## Results

Preliminary data indicate that the overall non-participation rate is 14.2%. The most common reasons for non-participation are treatment preconceptions (63.5%) and unwillingness to participate in research (25.4%). A minority of patients chose to have no treatment at all (6.3%). Incorporation of the video significantly reduced refusal rates from 21.1% to 8.5% (p<0.01).

## Conclusion

Treatment preconceptions are the predominant reason non-participation in a RCT. Incorporation of the video significantly reduced non-participation rates; it is possible that the involvement of the PI might have a positive effect on recruitment.

